# Honokiol blocks store operated calcium entry in CHO cells expressing the M3 muscarinic receptor: honokiol and muscarinic signaling

**DOI:** 10.1186/1423-0127-20-11

**Published:** 2013-02-23

**Authors:** Hsiu-Jen Wang, Alexis G Martin, Po-Kuan Chao, Rhett A Reichard, Adam L Martin, Yue-wern Huang, Ming-Huan Chan, Robert S Aronstam

**Affiliations:** 1 Department of Biological Sciences, Missouri University of Science & Technology, 400 W. 11th St, Rolla MO 65409, USA; 2 Department of Life Science, National Taiwan Normal University, 116, Taipei, Taiwan, R O C; 3 Institute of Neuroscience, National Chengchi University, Taipei, Taiwan

**Keywords:** Honokiol, Calcium signaling, Store operated calcium entry (SOCE), Inositol trisphosphate (IP3), Muscarinic acetylcholine receptor, Phospholipase Cβ

## Abstract

**Background:**

Honokiol, a cell-permeable phenolic compound derived from the bark of magnolia trees and present in Asian herbal teas, has a unique array of pharmacological actions, including the inhibition of multiple autonomic responses. We determined the effects of honokiol on calcium signaling underlying transmission mediated by human M3 muscarinic receptors expressed in Chinese hamster ovary (CHO) cells. Receptor binding was determined in radiolabelled ligand binding assays; changes in intracellular calcium concentrations were determined using a fura-2 ratiometric imaging protocol; cytotoxicity was determined using a dye reduction assay.

**Results:**

Honokiol had a potent (EC50 ≈ 5 μmol/l) inhibitory effect on store operated calcium entry (SOCE) that was induced by activation of the M3 receptors. This effect was specific, rapid and partially reversible, and was seen at concentrations not associated with cytotoxicity, inhibition of IP3 receptor-mediated calcium release, depletion of ER calcium stores, or disruption of M3 receptor binding.

**Conclusions:**

It is likely that an inhibition of SOCE contributes to honokiol disruption of parasympathetic motor functions, as well as many of its beneficial pharmacological properties.

## Background

Honokiol is a small, cell-permeable phenolic compound derived from the bark of magnolia trees that is present in Asian herbal teas. Honokiol has a unique array of antiangiogenic, antitumor and anti-inflammatory actions [1].

In the central and peripheral nervous systems, honokiol has a neuroprotective effect, as well as effects on certain signal transduction pathways. Honokiol is an effective scavenger of reactive oxygen species [2], which may be an important factor in its anti-inflammatory and neuroprotective actions [3-5]. Honokiol also has pronounced anti-nociceptive effects, notably the nociception associated with glutamate receptor activation [6,7]. Honokiol inhibits muscarinic receptor- and depolarization-mediated contraction of isolated porcine trachea, guinea pig ileum and rat uterus, apparently by blocking the influx of extracellular calcium [8-10]. Thus, common aspects of honokiol action in excitable tissues are an antioxidative action and depression of glutamate-receptor and calcium mediated signaling. It is possible that these are interrelated factors.

The present research was undertaken to determine the effects of honokiol on calcium signaling underlying transmission mediated by muscarinic receptors expressed in Chinese hamster ovary (CHO) cells, particularly receptor-mediated release from the endoplasmic reticulum through activated IP3 receptors and the subsequent store-operated calcium entry (SOCE) that is induced by depletion of calcium from the endoplasmic reticulum [11]. We report that honokiol is a potent inhibitor of SOCE, with lesser effects on resting calcium concentrations and calcium release from the endoplasmic reticulum.

## Methods

### Materials

**Figure 1:**
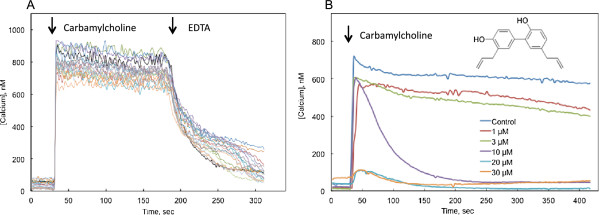
**Influence of honokiol on cellular responses to activation of M3 muscarinic receptors. **CHO cells expressing the human M3 receptor were loaded with fura-2 to detect changes in cytosolic calcium concentration. The cells were untreated (**A**) or treated with the indicated concentrations of honokiol for 10 min immediately prior to measuring calcium content (**B**). **A**: Control calcium responses to M3 receptor activation. Carbamylcholine (10 μmol/l) was added at the time indicated by the first arrow. This lead to a rapid and sustained increase in cytosolic calcium from 30 to 600-900 nmol/l. Addition of 5 mM EDTA to the medium (second arrow) decreased cytosolic calcium concentration. This is consistent with the interpretation that the sustained increase in calcium concentration following receptor activation is dependent on the influx of extracellular calcium through SOCE channels [12]. Each line represents the response of a single cell from typical experiment repeated over 100 times. **B**: Honokiol (inset) selectively depresses sustained calcium entry. The initial calcium response to receptor activation (arrow) was largely unaffected by honokiol at concentrations up to 20 μmol/l, but 10 μmol/l honokiol blocked the sustained entry, suggesting a selective blockade of SOCE. At higher concentrations both the initial and sustained responses were depressed. Each line represents the average response of 18-23 cells from a typical experiment; 4 experiments yielded essentially similar results.

Honokiol (2-(4-hydroxy-3-prop-2-enyl-phenyl)- 4-prop-2-enyl-phenol; Figure 1B) was synthesized and kindly provided by Dr. Chinpiao Chen, Department of Chemistry, National Dong Hwa University, Hualien, Taiwan.

### Cell culture and cytotoxicity measurements

CHO cells stably transfected with a clone of the human M3 muscarinic acetylcholine receptor (cDNA Resource Center, http://www.cdna.org) were cultured in DME/F12 media containing 10% FBS and penicillin/streptomycin (100 IU/100 μg/ml) at 37° in a 5% CO_2_ humidified atmosphere. Cell viability was determined using an MTS reduction assay (3-(4,5-dimethylthiazol-2-yl)-5-(3-carboxymethoxyphenyl)-2-(4-sulfophenyl)-2H-tetrazolium, inner salt; Promega, Madison). Cells (≈ 7,000) were seeded into each well of a 96-well plate and incubated for 16 h. The cells were exposed to honokiol for 24 h before cell viability was determined.

### Muscarinic receptor binding

[^3^H]*N*-methylscopolamine ([^3^H]MS; PerkinElmer), a non-selective muscarinic antagonist, was used to label muscarinic receptors from rat brain. Membranes from the neocortex were homogenized in 50 mmol/l Tris–HCl (pH 7.4), and an aliquot (15-30 μg protein) was incubated in the presence of [^3^H]MS and 50 mmol/l Tris–HCl (pH 7.4) for 60 minutes at room temperature in a final volume of 1 ml. Binding was determined by filtration of the suspension through glass fiber filters (1 μm pore size), followed by measurement of the radioactivity trapped on the filters by liquid scintillation counting. Non-specific binding was determined in the presence of 10 μmol/l atropine.

[^3^H]MS binding parameters (receptor concentration [Bmax] and dissociation constant [K_D_]) were determined by nonlinear regression (DeltaGraph) using a model for a single population of independent binding sites. Agonist binding was determined in competition studies using carbamylcholine and 0.2 nmol/l [^3^H]MS.

### Measurement of intracellular calcium

Cytosolic calcium concentration ([Ca^2+^]_i_) was measured in a ratiometric assay using the calcium-sensitive fluorescent dye, fura-2 (Molecular Probes). Cells (≈120,000) were seeded onto 29 mm glass bottom dishes and incubated for 16 h. The cells were then incubated with fura-2 AM for 60 min at room temperature in a basal salt solution (BSS) comprised of 130 mmol/l NaCl, 5.4 mmol/l KCl, 5.5 mmol/l glucose, 2 mmol/l CaCl_2_, 1 mmol/l MgCl_2_, and 20 mmol/l HEPES, at pH 7.4. The cells were then washed twice with BSS, and the incubation continued for an additional 30 min before measuring calcium levels. Honokiol was present during the last 10 min of the final incubation period.

[Ca^2+^]_i._ was determined at room temperature using an InCyt Basic IM™ Fluorescence Imaging System (Intracellular Imaging, Inc., Cincinnati, OH). The imaging system was calibrated for calcium concentration using standard calcium solutions of known concentration (Invitrogen #C3722), according to manufacturer protocol. The ratio of fluorescence intensities measured at 512 nm after excitation at 340 and 380 nm is a reliable indicator of [Ca^2+^]_i._.

Signals from 17-25 cells were measured in each plate. Cells in which the resting [Ca^2+^]_i._ was not maintained at a constant level (typically 1-2 cells/plate) were excluded from the analysis, as were cells that completely failed to respond to a muscarinic agonist (typically < 1 cell/plate). To measure responses in the absence of extracellular calcium, the cells were switched to calcium-free BSS 3 min before imaging.

### Statistical analysis

Parameters (calcium concentrations, dissociation constants, receptor concentrations, IC50’s) were compared as the means from 3-6 independent experiments (with 3 replicates in each binding measurement protocol and 17-23 replicates in each calcium measurement protocol) using Student’s *t*-test. Values from experiments with multiple independent variables were compared by ANOVA and Tukey’s test using GraphPad Prism software. Significant differences were indicated by P values of < 0.05.

## Results

### Honokiol inhibition of muscarinic signaling

The resting cytosolic calcium concentration ([Ca^2+^]_i_) of CHO-M3 cells in BSS in this series of experiments was 32.7 ± 9.8 nmol/l (mean ± S.D.; N = 5). Stimulation with the muscarinic agonist carbamylcholine (10 μmol/l) led to a rapid and sustained increase in cytosolic calcium to a concentration that ranged from 500 to 900 nmol/l (Figure 1A). The threshold concentration for elicitation of a response to carbamylcholine was approximately 0.2 μmol/l. Responses to carbamylcholine were completely blocked by a 10 min pretreatment with 10 μmol/l atropine, and CHO cells not transfected with a muscarinic receptor gene did not respond to carbamylcholine. Addition of EDTA (5 mmol/l) to the external medium caused a rapid decrease in [Ca^2+^]_i_, indicating that the sustained elevation of [Ca^2+^]_i_ following muscarinic receptor activation was dependent upon the entry of extracellular calcium (Figure 1A).

The initial change in [Ca^2+^]_i_ largely reflects calcium release from the endoplasmic reticulum, while the sustained plateau phase almost exclusively reflects calcium entry from the extracellular medium [12,13]. Initial calcium release from the ER is mediated by IP3 receptor ionophores, while the plateau phase involves “capacitive” calcium entry through Orai channels in the plasma membrane. STIM1 proteins span the ER membrane and possess a domain that senses calcium depletion. Calcium depletion induces an interaction between STIM1 and Orai, thereby activating the influx of extracellular calcium [13,14]. This influx of extracellular calcium in response to depletion of calcium in the endoplasmic reticulum is referred to as store-operated calcium entry (SOCE).

Honokiol blocked sustained calcium entry (i.e., SOCE) at concentrations at which it did not affect initial calcium release (i.e., calcium release from the ER (Figure 1B). For example, 10 μmol/l honokiol completely inhibited SOCE without significantly depressing the initial rise in [Ca^2+^]_i_. At higher concentrations (20 and 30 μmol/l), honokiol blocked both calcium responses.

**Figure 2:**
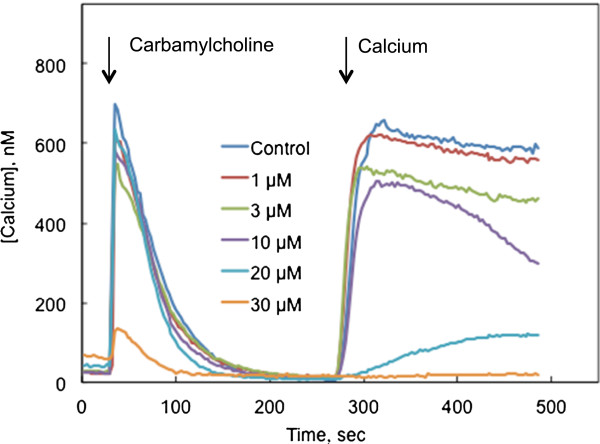
**Influence of honokiol on cellular responses to activation of M3 muscarinic receptors measured in the absence of extracellular calcium. **CHO cells expressing the human M3 receptor were loaded with fura-2 to monitor changes in cytosolic calcium concentration. The cells were untreated or treated with the indicated concentrations of honokiol for 10 min immediately before measuring calcium content. Cells were switched to a calcium-free BSS 3 min before imaging. Each line represents the average response from 17-24 cells; each panel presents the results from a separate experiment. Essentially similar results were obtained in three other experiments. Carbamylcholine (10 μmol/l) was added at the time indicated by the first arrow. Calcium was added to the extracellular medium (final concentration = 2 mmol/l) at the time indicated by the second arrow.

When the cells were switched to a calcium-free BSS 3 min before imaging, the initial response to carbamylcholine remained largely intact at honokiol concentrations up to 20 μmol/l (Figure 2A). These responses were not maintained in the absence of extracellular calcium, and cytosolic calcium concentrations declined to resting levels within a few minutes. At 30 μmol/l, however, honokiol severely depressed the response to carbamylcholine.

**Figure 3:**
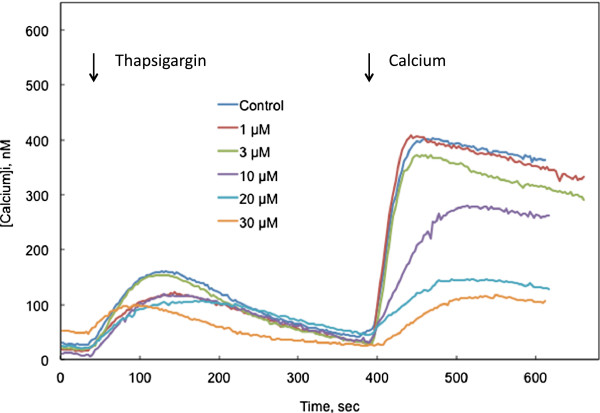
**Influence of honokiol on cytosolic calcium concentrations in calcium-free BSS in response to thapsigargin-induced depletion of calcium from the ER and to the subsequent reintroduction of extracellular calcium. **CHO-M3 cells were untreated or treated with various concentrations of honokiol, as indicated, for 10 min before measuring calcium content. The cells were switched to a calcium-free BSS 3 min before imaging. Thapsigargin (1 μmol/l) was added at the time indicated by the first arrow. Calcium was added to the extracellular medium (final concentration = 2 mmol/l) at the time indicated by the second arrow. Each line represents the average from four experiments, each with 15-22 cells per measurement.

SOCE can also be observed following depletion of ER calcium stores in the absence of extracellular calcium. This depletion can be accomplished by stimulation of muscarinic receptors (Figure 2) or by treating with thapsigargin (1 μmol/l) to inhibit the sarco/endoplasmic reticulum calcium-ATPases (SERCA) that transport calcium into the ER against a large concentration gradient (Figure 3). In response to this depletion, SOCE channels open, although calcium is not available to enter the cell. When [Ca^2+^]_i_ levels return to a background level, influx through SOCE channels can be visualized by adding calcium to the extracellular medium (2 mmol/l final concentration). These methods reveal a potent inhibitory effect of honokiol on SOCE. Again, inhibition of SOCE is seen at honokiol concentrations that do not affect release of calcium from the ER in response to carbamylcholine or thapsigargin (Figures 2 &3). Honokiol had a somewhat inconsistent effect on thapsigargin-induced calcium release from the ER, depressing the rise in [Ca^2+^]_i_ in some experiments (e.g., Figure 3), but not in others. However, in all cases, SOCE was more sensitive to honokiol inhibition than ER-mediated calcium release.

**Figure 4:**
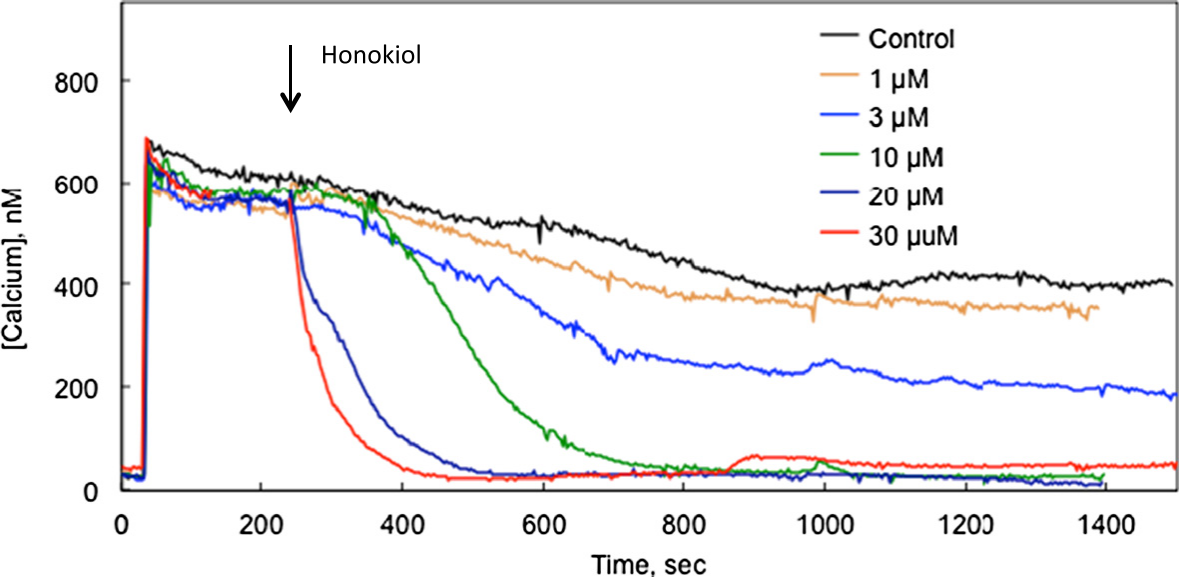
**Acute effect of honokiol on SOCE. **CHO-M3 cells were stimulated with 10 μmol/l carbamylcholine at 40 sec. Honokiol was added to the extracellular medium at the time indicated by the arrow. Each line represents the average from 19-21 cells; three other experiments yielded essentially similar results.

In one series of experiments, the acute effects of honokiol were determined by stimulating CHO-M3 cells with a maximally effective concentration of carbamylcholine (10 μmol/l) followed by exposing the cells to honokiol during the sustained phase of calcium entry (i.e., during SOCE). Calcium entry was blocked by honokiol in a concentration-dependent manner (Figure 4). The threshold for this inhibition was between 1 and 3 μmol/l.

**Figure 5:**
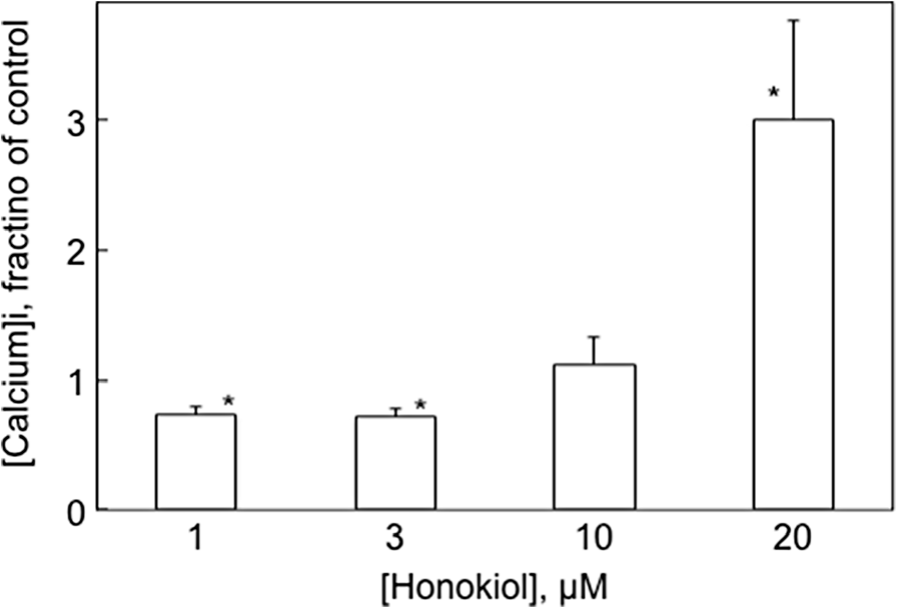
**Influence of honokiol on resting calcium concentrations in CHO-M3 cells. **This figure summarizes the effects of a 10 min exposure to honokiol at the indicated concentrations on the initial resting calcium concentration. Calcium concentrations are expressed as fraction of the calcium concentration in control cells not exposed to honokiol in the same experiment. Significant differences in the calcium concentrations of treated cells compared to control cells are indicated by the asterisks (p < 0.05; ANOVA and Tukey’s test; N = 10).

Honokiol had a biphasic effect on resting [Ca^2+^]_i_ level. Results gleaned from all of the experiments described in this paper are summarized in Figure 5. At 1 and 3 μmol/l, honokiol decreased [Ca^2+^]_i_ by about 20%, but at 20 μmol/l honokiol caused a large and variable increase in [Ca^2+^]_i_.

### Honokiol cytotoxicity

**Figure 6:**
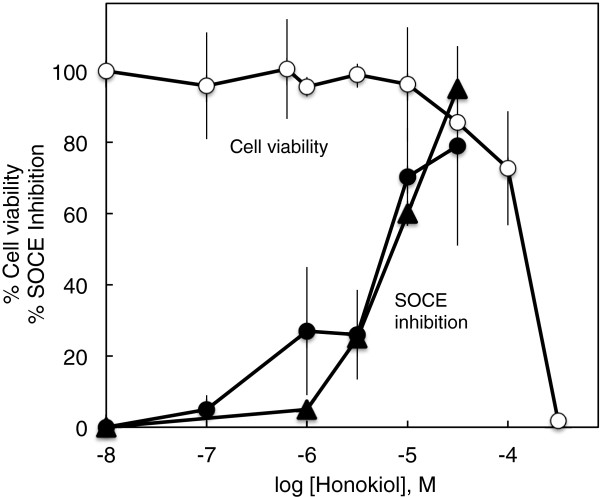
**Cytotoxicity of honokiol and comparison to honokiol inhibition of SOCE. **The cytotoxicity of honokiol following a 24 h exposure period is presented as fractional decrease in cells capable of maintaining an intracellular reducing environment (open circles). Fractional inhibition of SOCE by a 10 min exposure to honokiol was determined in two ways: 1) depression of sustained calcium entry (●) (e.g., Figure 1) and 2) inhibition of calcium influx following thapsigargin depletion of ER calcium stores (▲) (e.g., Figure 3).

CHO-M3 cell viability was revealed by the ability of CHO cells to reduce MTS. Exposure of CHO cells to honokiol for 24 h induced cytotoxicity in a concentration-dependent manner (Figure 6). Cytotoxicity was not observed with honokiol concentrations up to 32 μmol/l. Cytotoxicity was 20% with 100 μmol/l honokiol and complete with 320 μmol/l honokiol. It is significant that a 10 min exposure to honokiol inhibited SOCE at concentrations (e.g., 1 - 30 μmol/l) that did not cause cytotoxicity following a 24 h exposure. In Figure 6, dose response relationships for honokiol inhibition of SOCE are compared to the 24 h cytotoxicity dose–response relationship. SOCE inhibition by a 10 min exposure to honokiol was determined in two ways: 1) depression of sustained calcium entry induced by carbamylcholine (e.g., Figure 1B); and 2) inhibition of calcium influx following thapsigargin depletion of ER calcium (e.g., Figure 3). The EC50s for honokiol inhibition of SOCE (10 min) were an order of magnitude lower than the EC50 for honokiol cytotoxicity (24 h), and the EC50s for the two measures of honokiol inhibition of SOCE were similar.

### Reversibility of honokiol effects on muscarinic signaling

**Figure 7:**
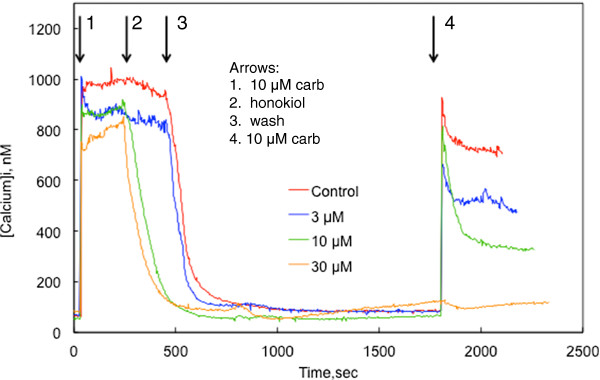
**Reversibility of honokiol effects on calcium signaling. **Carbamylcholine (10 μmol/l) was added at the point indicated by the first arrow. Honokiol (3, 10 or 30 μmol/l, as indicated) was added at the time indicated by the second arrow. The cells were washed 3 times over a 2 min period with fresh media, beginning at the time indicated by the third arrow. The cells were again stimulated with carbamylcholine (10 μmol/l) at the time indicated by the fourth arrow. The data is the average from 19-22 cells from a representative experiment performed 3 times with essentially similar results.

The persistence of honokiol inhibition of SOCE is revealed by the experiments depicted in Figure 7. After cells had reached a plateau level of SOCE, honokiol was added to the extracelluluar medium. High concentrations of honokiol (10 and 30 μmol/l) inhibited calcium entry, while 3 μmol/l honokiol did not. The cells were washed 3 times with fresh buffer to remove both the honokiol and carbamylcholine. Within 10 min a stable baseline [Ca^2+^]_i._ was obtained. After about 20 min, the cells were again stimulated with 10 μmol/l carbamylcholine. The initial response of control cells to the second application of carbamylcholine was not affected, although [Ca^2+^]_i._ relaxed to a plateau level that was 20% lower than the first plateau level. This suggests a desensitization of a portion of the response. In cells exposed to 3 and 10 μmol/l honokiol, however, the initial response to the second application of carbamylcholine was somewhat diminished (10-25%), while the sustained plateau levels were severely depressed (i.e., by 50-70%). This is consistent with a specific effect of honokiol on SOCE that is only partially reversible. In contrast, cells exposed to 30 μmol/l honokiol failed to response to the second application of carbamylcholine.

### Influence of honokiol on ligand binding to muscarinic receptors

**Figure 8:**
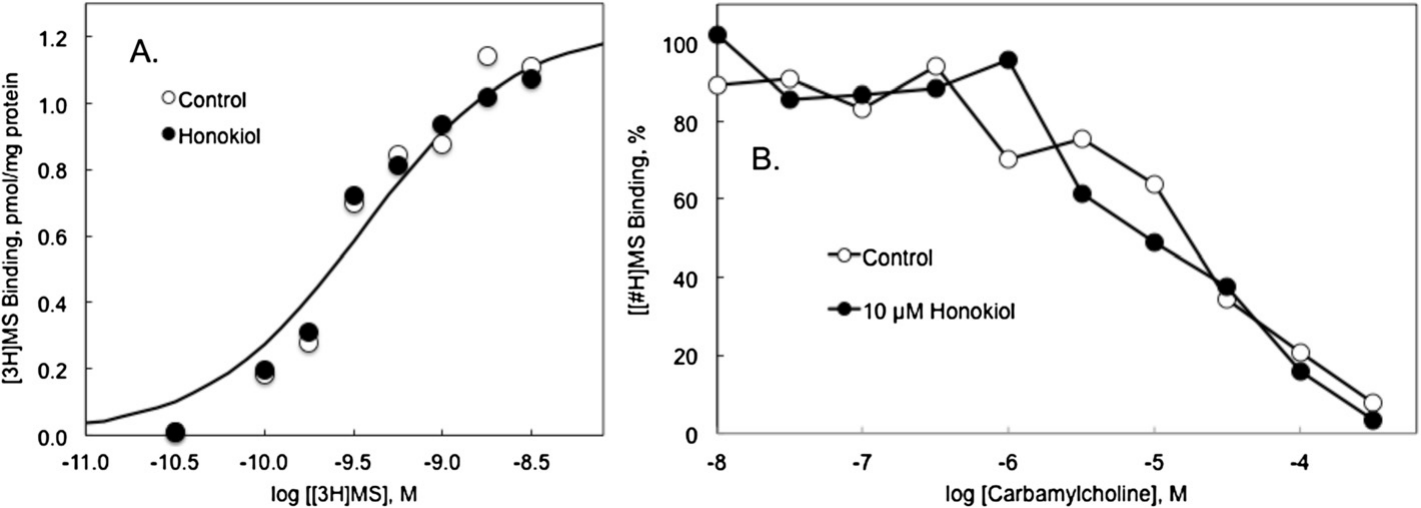
**Influence of honokiol on ligand binding to muscarinic receptors in rat neocortical membranes. A**: The binding of [^3^H]MS was determined in the absence) open circles) and presence (closed circles) of 10 μmol/l honokiol. Lines are drawn according to nonlinear regression to a model incorporating a single population of independent binding sites that revealed the following binding parameters in the absence and presence of honokiol, respectively: Bmax = 1.3 ± 0.2 and 1.2 ± 0.2 pmol/mg; KD = 0.40 ± 0.03 and 0.35 ± 0.5 nmol/l; N = 3). **B**: Influence of honokiol on agonist binding to neural muscarinic receptors revealed by the ability of carbamylcholine to inhibit the specific binding of 0.2 nmol/l [^3^H]MS.

The binding of [^3^H]MS to brain muscarinic receptors is shown in Figure 8. Brain membranes were used as an abundant source of muscarinic receptors. Approximately 40% of the neocortical receptors are of the M3 subtype. Saturation binding curves were performed in the absence and presence of 10 μmol/l honokiol. Honokiol did not affect the apparent concentration of receptors (Bmax = 1.3 ± 0.2 and 1.2 ± 0.2 pmol/mg protein in the absence and presence of honokiol, respectively) or the affinity of [^3^H]MS for the receptors (0.40 ± 0.03 and 0.35 ± 0.5 nmol/l, respectively) (Figure 8A). Agonist binding to muscarinic receptors is sensitive to multiple aspects of receptor organization, including coupling to G proteins. However, agonist binding, as revealed by the ability of carbamylcholine to inhibit the binding of 0.3 nmol/l [^3^H]MS to the receptor was not affected by 10 μmol/l honokiol (Figure 8B).

## Discussion

### Muscarinic signaling in CHO cells

The influence of honokiol on muscarinic signaling was investigated in CHO cells expressing the human M3 muscarinic receptor. In wild-type CHO cells, we cannot detect acetylcholinesterase activity, muscarinic receptor binding, or muscarinic signaling as reflected in either calcium mobilization or alteration of cAMP synthesis in response to carbamylcholine. However, CHO cells express components of both the phospholipase Cβ [15] and adenylate cyclase [16] signaling pathways, and CHO cells transfected with transgenes for the different muscarinic receptor subtypes respond to muscarinic agonists in pharmacologically and physiologically appropriate fashions: Activation of human M1, M3 or M5 receptors expressed in CHO cells leads to the production of IP3, release of calcium from the ER, activation of SOCE, and modulation of the expression of genes under control of the AP-1 or NFAT response elements [15] (AL Martin et al., unpublished observations). Activation of human M2 and M4 receptors expressed in CHO cells leads to the inhibition of forskolin-stimulated cAMP formation and alteration of the expression of genes under the control of the cAMP response element (CRE) [15] (AL Martin et al., unpublished observations).

M3 receptors activate Gα_q_ transducer proteins that stimulate phospholipase Cβ (PLCβ) [17]. PLCβ hydrolyzes phosphatidylinositol 4.5-bisphosphate, releasing both diacylglycerol and inositol trisphosphate (IP3). IP3 binds to the IP3 receptor/ionophore on the endoplasmic reticulum (ER) to release calcium into the cytosol. Sensors in STIM1 proteins that span ER membranes are sensitive to the depletion of ER calcium. This leads to a close apposition of ER and plasma membranes, allowing STIM1 proteins to interact with Orai channel proteins in the plasma membrane, thereby stimulating the entry of extracellular calcium. This is called capacitive calcium entry or store-operated calcium entry (SOCE) [11,13,18].

### Honokiol disruption of muscarinic signaling

Calcium release from the ER through the IP3 receptor ionophore was visualized as the initial release of calcium in response to carbamylcholine. This release was not affected by honokiol in either the presence or absence of extracellular calcium (Figures 1 &2). Honokiol had variable effects on calcium release from the ER induced by thapsigargin. In multiple instances, honokiol did not affect thapsigargin-induced release at concentrations that produced strong inhibition of SOCE. However, at higher concentrations, an inhibition of both thapsigargin-induced release and SOCE was observed. The precise relationship between the thapsigargin-induced release, ER calcium concentration, and SOCE gating requires additional clarification.

SOCE was visualized by depleting calcium from the ER by stimulating the muscarinic-IP3 receptor pathway or by inhibiting SERCA pumps in the absence of extracellular calcium. When [Ca^2+^]_i_ returned to its baseline level (3-5 min), calcium was added to the extracellular medium (final concentration = 2 mmol/l). Calcium immediately entered the cell via the SOCE channels that had opened in response to depletion of ER calcium. In both paradigms, honokiol displayed a strong inhibition of extracellular calcium entry through SOCE channels.

Honokiol’s effect on SOCE was selective insofar as honokiol inhibited SOCE at lower concentrations than it affected ER calcium release induced by either IP3 receptor activation or thapsigargin inhibition of SERCA. Honokiol’s effect was rapid and partially reversible (at concentrations below 30 μmol/l), and was observed at concentrations and under conditions in which honokiol was not cytotoxic and did not interfere with ligand binding to the muscarinic receptor. Previous work has documented honokiol inhibition of muscarinic receptor- and depolarization-mediated contraction of isolated porcine trachea, guinea pig ileum and rat uterus, apparently by blocking the influx of extracellular calcium [8-10]. The present work suggests that an inhibition of SOCE is the mechanism underlying these effects on autonomic signaling.

The threshold for honokiol inhibitory effects *in vitro* was ≈ 3 μM. This is consistent with the concentration dependence of *in vitro* antiinflammatory, antithrombosis, signaling and antioxidant effects of honokiol. For example, Alexeev et al. [19] noted magnonol and honokiol effects on GABAergic transmission in the 1-30 μM range, Tian et al. used 150 μM honkiol to enhance cell death of cultured cancer cells [20], Nagalingam et al. found that honokiol concentrations of 5 μM honokiol were required to inhibit the clonogenicity of cultured breast cancer cells by activation of AMP-activated protein kinase [21], and Fukuyama et al., demonstrated a neurtorphic acivity (neurtie outgrowth) of 0.1 – 10 μM honokiol on cultured rat cortical neurons [22]. Moreover, Lu et al. used 1 -100 μM honokiol to inhibit responses of uteri to cholinergic agonists [10] and Ko et al used 0.1 -100 μM honokiol to inhibited cholinergic responses of tracheal muscle [9].

In earlier work we demonstrated that oxidative stress (e.g., by exposure to metal oxide nanoparticles) also has a selective inhibitory effect on SOCE [15]. Honokiol, however, has antioxidant properties and is cytoprotective in the face of multiple insults, including oxidative stress. This raises the possibility that honokiol actions reflect a direct interaction with the ion channel (Orai) itself, rather than an indirect effect on the redox state of the cell. This would be consistent with honokiol’s antioxidant activity, lack of cytotoxicity, and rapid and partially reversible inhibition of SOCE. Another possibility that remains to be evaluated is whether honokiol disrupts Orai – Stim1 interactions. It is interesting that honokiol, unlike most pharmacologically exploited ion channel blockers, is not a hydrophobic amine.

The effect of honokiol on resting [Ca^2+^]_i._ was biphasic. The decrease in [Ca^2+^]_i._ at honokiol concentrations below 10 μmol/l might reflect the role of Orai channels as one of the transport mechanisms that determine cellular calcium concentration [23]. It is also possible that honokiol alters the resting calcium content aof the endoplasmic reticulum, which might reflect changes in active sequestration mechanisms (SERCA) or ER channel conductances. Increases in resting [Ca^2+^]_i._ are associated with incipient cytotoxicity. Honokiol recently was reported to promote calcium release associated with mitochondrial dysfunction in human chondrosarcoma cells [24]. However, even at high and ultimately cytotoxic concentrations of honokiol, virtually all of the cells remained capable of maintaining [Ca^2+^]_i_ at constant levels over the time course of these acute experiments.

## Conclusions

Honokiol had a potent inhibitory effect on store operated calcium entry (SOCE) stimulated by activation of the M3 muscarinic receptors. This effect was specific, rapid and partially reversible, and was seen at concentrations not associated with cytotoxicity, inhibition of IP3 receptor-mediated release, or disruption of M3 receptor binding. It is likely that inhibition of SOCE accounts for honokiol disruption of parasympathetic motor functions, and may contribute to certain of its beneficial pharmacological properties.

## Abbreviations

BSS: Basal salt solution; [Ca2+]i: Intracellular calcium concentration; CHO: Chinese hamster ovary; ER: Endoplasmic reticulum; IP3: Inositol trisphosphate; [3H]MS: [^3^H]*N*-methylscopolamine; MTS: 3-(4,5-dimethylthiazol-2-yl)-5-(3-carboxymethoxyphenyl)-2-(4-sulfophenyl)-2H-tetrazolium; PLCβ: Phospholipase Cβ; SERCA: Sarco/endoplasmic reticulum calcium-ATPase; SOCE: Store operated calcium entry

## Competing interests

The authors declare that they have no competing interests.

## Authors’ contributions

H-JW measured calcium responses and cell viability and revised the manuscript. AGM, P-KC and ALM measured calcium responses, performed data analysis and reviewed the manuscript. RAR performed the ligand binding measurements, performed data analysis and reviewed the manuscript. Y-WH was involved in study design and preparing the manuscript. M-HC was involved in study design, data interpretation and manuscript review. RSA designed the experiments, analyzed the data and prepared the manuscript. All authors read and approved the final manuscript.

## Authors’ information

Co-Corresponding author: Ming-Huan Chan, Ph.D. Institute of Neuroscience, Research Ctr for Mind, Brian & Learning, National, Chengchi University, No. 64, Sec 2, ZhiNan Rd. Taipei, Taiwan.
